# The μ opioid agonist morphine modulates potentiation of capsaicin-evoked TRPV1 responses through a cyclic AMP-dependent protein kinase A pathway

**DOI:** 10.1186/1744-8069-2-22

**Published:** 2006-07-16

**Authors:** Irina Vetter, Bruce D Wyse, Gregory R Monteith, Sarah J Roberts-Thomson, Peter J Cabot

**Affiliations:** 1 The School of Pharmacy, The University of Queensland, Brisbane, 4072, Australia

## Abstract

**Background:**

The vanilloid receptor 1 (TRPV1) is critical in the development of inflammatory hyperalgesia. Several receptors including G-protein coupled prostaglandin receptors have been reported to functionally interact with the TRPV1 through a cAMP-dependent protein kinase A (PKA) pathway to potentiate TRPV1-mediated capsaicin responses. Such regulation may have significance in inflammatory pain. However, few functional receptor interactions that inhibit PKA-mediated potentiation of TRPV1 responses have been described.

**Results:**

In the present studies we investigated the hypothesis that the μ opioid receptor (MOP) agonist morphine can modulate forskolin-potentiated capsaicin responses through a cAMP-dependent PKA pathway. HEK293 cells were stably transfected with TRPV1 and MOP, and calcium (Ca^2+^) responses to injection of the TRPV1 agonist capsaicin were monitored in Fluo-3-loaded cells. Pre-treatment with morphine did not inhibit unpotentiated capsaicin-induced Ca^2+ ^responses but significantly altered capsaicin responses potentiated by forskolin. TRPV1-mediated Ca^2+ ^responses potentiated by the direct PKA activator 8-Br-cAMP and the PKC activator Phorbol-12-myristate-13-acetatewere not modulated by morphine.

Immunohistochemical studies confirmed that the TRPV1 and MOP are co-expressed on cultured Dorsal Root Ganglion neurones, pointing towards the existence of a functional relationship between the G-protein coupled MOP and nociceptive TRPV1.

**Conclusion:**

The results presented here indicate that the opioid receptor agonist morphine acts via inhibition of adenylate cyclase to inhibit PKA-potentiated TRPV1 responses. Targeting of peripheral opioid receptors may therefore have therapeutic potential as an intervention to prevent potentiation of TRPV1 responses through the PKA pathway in inflammation.

## Background

Peripheral sensitization can contribute to the development of persistent pain and involves numerous cellular processes. An important receptor in this process is the vanilloid receptor 1 or TRPV1. The TRPV1 is a nociceptive calcium (Ca^2+^) channel that is activated by capsaicin, the pungent constituent of chilli peppers, as well as protons and heat [1]. The TRPV1 is mainly expressed on nociceptive peripheral neurones and appears to be critical in the development of inflammatory hyperalgesia [1,2], as axonal transport of TRPV1 mRNA as well as TRPV1 protein expression are significantly increased in inflamed tissues [3,4], while mice lacking the TRPV1 develop less thermal hyperalgesia [2] and TRPV1 antagonists reversed thermal hyperalgesia in inflammation [5].

Phosphorylation of the TRPV1 by numerous kinases, including cAMP-dependent protein kinase A (PKA), can regulate function of the receptor [6-8]. cAMP levels are elevated in inflamed tissues [9,10] and the cAMP/PKA pathway appears to be essential in sensitizing inflammatory nociception and contributes to the development of inflammatory hyperalgesia induced by proinflammatory mediators such as prostaglandin E2 (PGE_2_) [9,11]. In line with reported sensitization of the TRPV1 in such inflammatory conditions [3,4], PKA-mediated phosphorylation of the TRPV1 occurs in neurones as well as heterologous expression systems treated with adenylate cyclase activators such as forskolin, or direct PKA activators such as 8-Br-cAMP; while PKA is also involved in PGE_2_- and anandamide-mediated TRPV1 sensitization as well as potentiation of capsaicin responses through the metabotropic glutamate receptor 5 [6,7,12-17]. Although sensitization of TRPV1 responses is important, the concept of preventing sensitization may also be important in modulating signalling through the TRPV1. However, few studies have investigated pathways that interact with the TRPV1 to prevent sensitization [12,14].

As a prototypical G-protein coupled receptor that produces analgesic effects upon activation [18], one such potential modulator is the μ opioid receptor or MOP. Direct G-protein coupled effects of opioid receptors include activation of inwardly rectifying potassium channels, inhibition of voltage-dependent Ca^2+ ^channels as well as inhibition of adenylate cyclase [18]. The present study describes the potential role of opioids, in particular the MOP agonist morphine, as modulators of TRPV1 responses by utilizing a HEK293 (human embryonic kidney) cell line stably expressing both TRPV1 and MOP and assesses the possible role of PKA in this modulation. Morphine did not affect unpotentiated TRPV1 responses nor TRPV1 responses potentiated by the direct PKA activator 8-Br-cAMP or the protein kinase C (PKC) activator Phorbol-12-myristate-13-acetate. However, TRPV1 responses potentiated by forskolin were modulated by morphine, highlighting the potential for endogenous opioid modulation of TRPV1 responses potentiated by the PKA pathway in inflammation.

## Results

### FLAG-MOP/TRPV1 double stable HEK293 cells express functional TRPV1 and MOP

**Figure 1:**
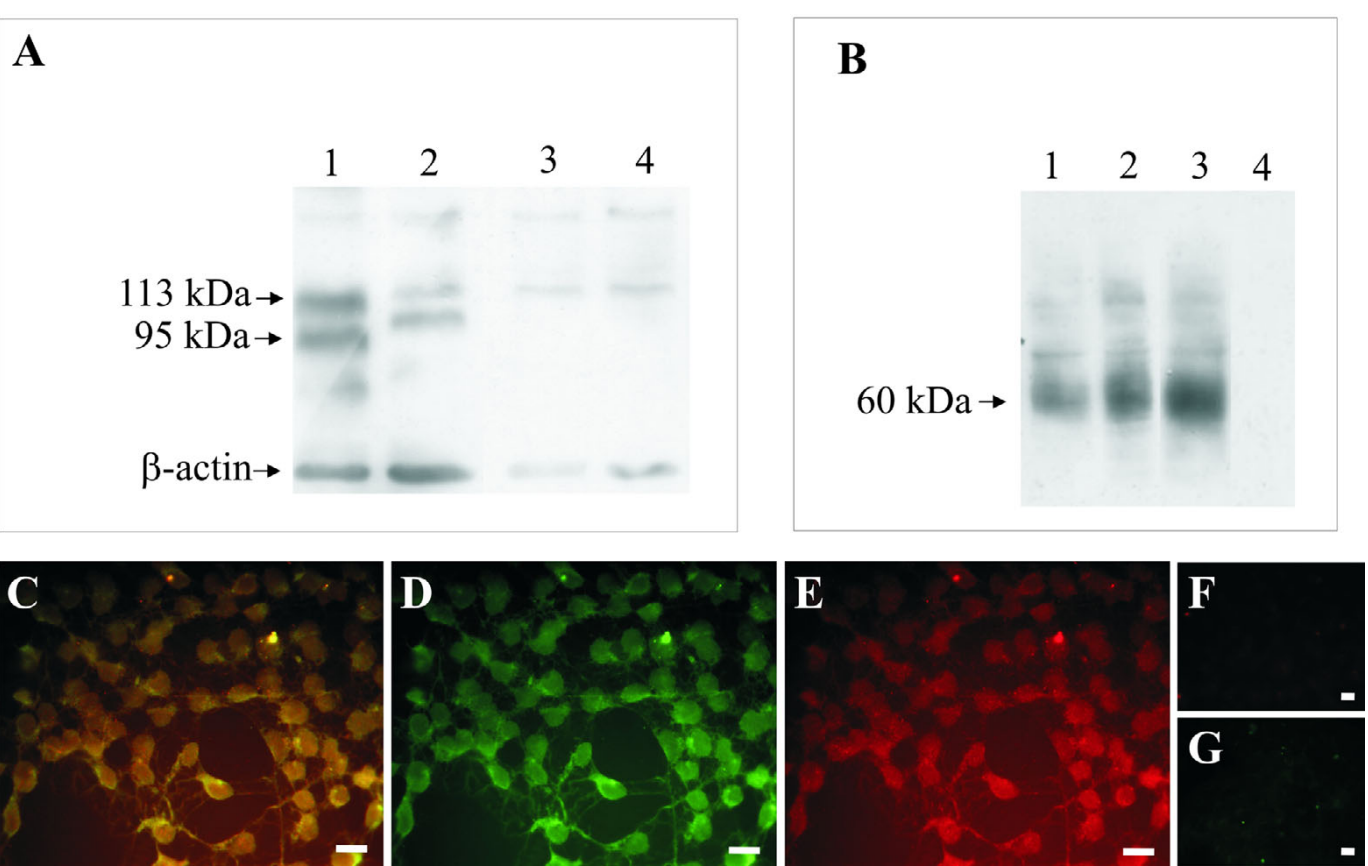
FLAG-MOP/TRPV1 double stable HEK293 cells express TRPV1 and MOP. (**A**) Western Blot Analysis of TRPV1 expression from HEK293 cells stably expressing TRPV1 and FLAG-tagged MOP. Precipitated cell proteins were subjected to SDS PAGE on a 7.5% gel, transferred to nitrocellulose membrane and probed for TRPV1 expression using a N-terminal directed TRPV1 antibody. β-actin was used as a loading control. Expected bands for the TRPV1 are at approximately 95 kDa for the unglycosylated and 113 kDa for the glycosylated receptor. Lane 1: Colony 13 FLAG-MOP/TRPV1 double expressant; Lane 2: Colony 21 FLAG-MOP/TRPV1 double expressant; Lane 3: untransfected HEK293 cells; Lane 4: FLAG-MOP only transfected HEK293 cells. (**B**) Western Blot showing FLAG-MOP protein in the same FLAG-MOP/TRPV1 and FLAG-MOP only cells, determined using an anti-FLAG M2 antibody. Expected protein size was a broad band at approximately 60 kDa representing multiple glyocsylated FLAG-MOP species. Lane 1: Colony 13 FLAG-MOP/TRPV1 double expressant; Lane 2: Colony 21 FLAG-MOP/TRPV1 double expressant; Lane 3: FLAG-MOP only transfected HEK293 cells; Lane 4: untransfected HEK293 cells. Representative blots from at least 3 independent experiments are presented. (**C–E**) FLAG-MOP/TRPV1 cells co-express TRPV1 and FLAG-MOP. Double stable FLAG-MOP/TRPV1 cells were stained for FLAG-MOP (**D**, green) and TRPV1 (**E**, red) expression with the overlay (**C**, yellow) clearly demonstrating co-expression of the two receptors. Specificity of the antibodies used was demonstrated by lack of non-specific binding (**F**, red and **G**, green). Scale bar, 40 μm.

**Figure 2:**
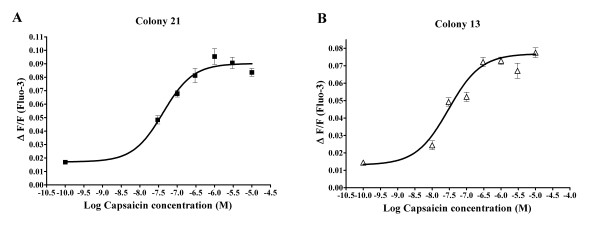
Capsaicin dose-response curves from two independent FLAG-MOP/TRPV1 expressants. Ca^2+ ^responses of Fluo-3-loaded cells to injection of capsaicin were measured using a fluorescent Microplate reader and maximum change in fluorescence, expressed as ΔF/F, was plotted as a function of capsaicin concentration. A 4-parameter Hill function was fitted to the data using GraphPad Prism (San Diego, California). (**A**) Capsaicin dose-response from FLAG-MOP/TRPV1 double expressant colony 13 (■). (**B**) Capsaicin dose-response from FLAG-MOP/TRPV1 double expressant colony 21 (△). Data are expressed as mean ± SEM with n = 8.

Receptor mRNA for both TRPV1 and FLAG-MOP was effectively translated to receptor protein as Western Blot analysis showed bands of the expected sizes for both receptors (Figure 1A and 1B). Two different colonies of FLAG-MOP/TRPV1 double stable HEK293 cells, colony 13 and colony 21, were selected for functional studies based on their expression of TRPV1 and FLAG-MOP protein (Figure 1A and 1B, lanes 1 and 2) and capsaicin responses (Figure 2). For both FLAG-MOP/TRPV1 colonies, the TRPV1 was present in two forms; a non-glycosylated form with a corresponding band at approximately 95 kDa, and a glycosylated form with a predicted molecular weight of 113 kDa (Figure 1A, lanes 1 and 2). Colony 13 (Figure 1A, lane 1) expressed slightly more TRPV1 than colony 21 (Figure 1A, lane 2) while no TRPV1 protein was detected in either untransfected HEK293 cells (Figure 1A, lane 3) or HEK293 cells that were transfected with FLAG-MOP only (Figure 1A, lane 4). Colonies 13 and 21 were selected for functional studies as they responded reproducibly to stimulation with capsaicin as described below (Figure 2). Untransfected HEK cells did not respond to stimulation with capsaicin (10 μM) (ΔF/F 0.010 ± 0.0015).

Colony 13 (Figure 1B, lane 1), colony 21 (Figure 1B, lane 2) as well as HEK293 cells transfected with FLAG-MOP only (Figure 2B, lane 3) showed a protein band at approximately 60 kDa, representative of numerous glycosylated forms of the MOP. Non-transfected HEK293 cells lacked this band (Figure 1B, lane 4). TRPV1 and MOP expression were also confirmed using immunohistochemistry with the overlay (Figure 1C, yellow) clearly demonstrating co-expression of the MOP (Figure 1D, green) and TRPV1 (Figure 1E, red). Non-specific binding is represented in Figures 1F and 1G for TRPV1 and MOP, respectively.

To assess functionality of the TRPV1 in the double stable cell lines, Ca^2+ ^responses to capsaicin were recorded. Both colonies 13 and 21 responded with concentration-dependent increases in intracellular free Ca^2+ ^to capsaicin (Figure 2). EC50s of approximately 34 nM and 40 nM were determined for colonies 13 and 21, respectively, similar to previous studies in TRPV1-transfected HEK293 cell lines [19-21]. The response to capsaicin was mediated through activation of the TRPV1, as the TRPV1 antagonist capsazepine (10 μM; Sigma) inhibited capsaicin responses to 6.4 ± 2.5% of the control response at 60 seconds.

### Morphine inhibits FSK-stimulated cAMP production in stable FLAG-MOP/TRPV1 HEK293 cell lines

**Figure 3:**
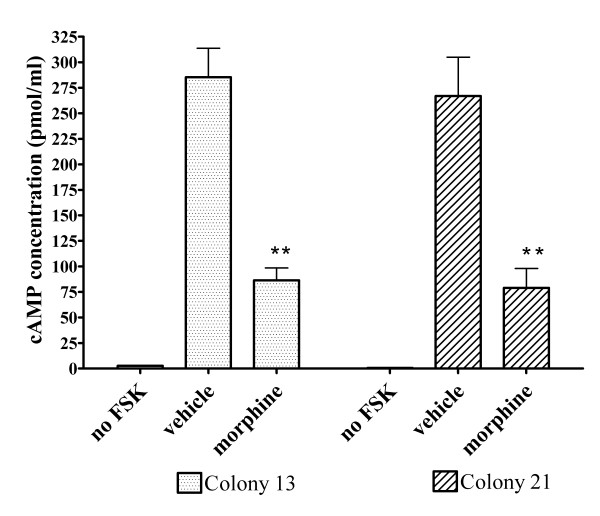
Inhibition of FSK-stimulated cAMP accumulation by morphine (1 μM). cAMP levels in FLAG-MOP/TRPV1 double stable colonies were measured using an enzyme-immunoassay kit either in unstimulated cells (no FSK), or in cells treated with FSK (50 μM) and IBMX (100 μM). Morphine (1 μM) significantly reduced cAMP levels in FSK-stimulated cells (morphine) compared to stimulated cells treated with water (vehicle). Morphine inhibited cAMP production by 67.0 ± 4.6% for FLAG-MOP/TRPV1 colony 13 (dotted bars) and 79.2 ± 7.4% for colony 21 (cross-hatched bars). Data are presented as mean ± SEM from n = 3 samples read in triplicate. ** *p *< 0.01 compared to vehicle.

Functionality of the FLAG-MOP was confirmed using a cAMP accumulation assay, which assesses functional coupling of the MOP to G_i _proteins [18]. Incubation with the phosphodiesterase inhibitor IBMX (100 μM) and the adenylate cyclase activator FSK (50 μM) significantly (*p *< 0.01) increased intracellular cAMP levels in both FLAG-MOP/TRPV1 colonies (Figure 3, vehicle) compared to untreated cells (Figure 3, no FSK). As expected, the MOP agonist morphine (1 μM) significantly (*p *< 0.01) inhibited the FSK-induced increased in cAMP production by 67.0 ± 4.6% for colony 13 and 79.2 ± 7.4% for colony 21, although cAMP levels were not reduced to unstimulated levels (Figure 3, morphine).

### Morphine reduces TRPV1-mediated capsaicin-induced Ca^2+ ^in the presence of FSK through activation of MOP

**Figure 4:**
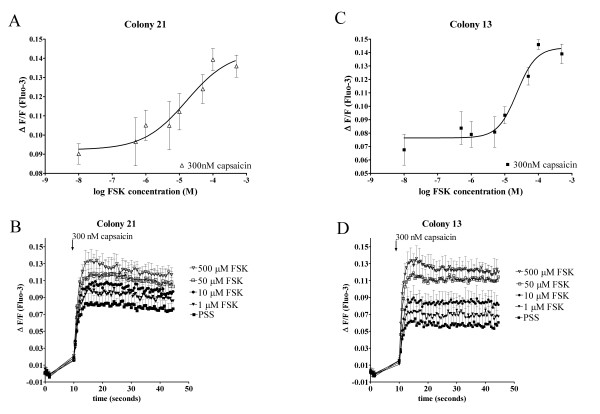
FSK potentiates capsaicin-induced Ca^2+ ^responses in FLAG-MOP/TRPV1 double stable HEK293 cells. Pre-incubation with varying concentrations of FSK (0.5–500 μM) and constant concentrations of IBMX (100 μM) caused a FSK-concentration-dependent increase in Ca^2+ ^response to the injection of 300 nM capsaicin in colony 21 (△(**A**)) and colony 13 (■, (**C**)). (**B **and **D**) Individual Ca^2+ ^traces after injection of 300 nM capsaicin from Fluo-3-loaded double colonies (Colony 21, (**B**) and Colony 13, (**D**)). Cells responded with a characteristic increase in intracellular Ca^2+ ^after injection of capsaicin, which was increased after 15 minutes incubation with FSK and IBMX (100 μM). Data are presented as mean ± SEM with n = 4.

We examined whether morphine can inhibit TRPV1-mediated increases in intracellular free Ca^2+ ^under basal conditions and with prior sensitization of TRPV1. To identify if cAMP is a potentiator of TRPV1 responses in our heterologous expression system, FLAG-MOP/TRPV1 transfected HEK293 cells were pre-incubated with varying concentrations of FSK as well as a constant concentration of IBMX (100 μM) and the Ca^2+ ^response to 300 nM capsaicin was determined (Figure 4). FSK activates adenylate cyclase, which in turn increases cellular cAMP levels, thus, FSK indirectly activates cAMP-dependent PKA. Pre-incubation with vehicle (DMSO) had no effect on Ca^2+ ^responses of colonies 13 (Figure 4A and 4B) and 21 (Figure 4C and 4D) to 300 nM capsaicin but increasing FSK concentrations potentiated capsaicin responses in a concentration-dependent manner (Figure 4A–D). Injection of FSK (50 μM) and IBMX (100 μM) caused a small (ΔF/F 0.0152 ± 0.0011) addition response that returned to baseline after approximately 3 s. This response was not affected (*p *> 0.05) by pre-incubation with morphine (1 μM) (*p *> 0.05; ΔF/F 0.0144 ± 0.0013).

**Figure 5:**
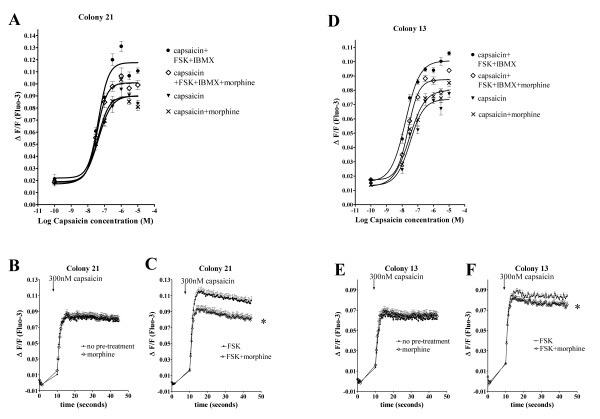
Morphine inhibits FSK-potentiated capsaicin responses in FLAG-MOP/TRPV1 double stable HEK293 cells but not capsaicin responses in absence of FSK. Double stable FLAG-MOP/TRPV1 colonies were loaded with Fluo-3 and pre-incubated with PSS for untreated or morphine (1 μM) for treated cells for 15 min followed by further incubation with buffer containing either no added drugs for unstimulated cells or FSK (50 μM) and IBMX (100 μM) stimulated cells. Ca^2+ ^responses after injection of capsaicin were monitored for 45 s using a fluorescent microplate reader. (**A **and **D**) Capsaicin dose-response curves for colony 21 (**A**) and colony 13 (**D**) without pre-treatment (capsaicin, ▼), after morphine incubation (capsaicin+morphine, **x**), after pre-incubation with FSK+IBMX (capsaicin+FSK+IBMX, ●) and after treatment with FSK, IBMX and morphine (capsaicin+FSK+IBMX+morphine, ◊). A 4-parameter Hill equation was fitted using GraphPad Prism. Data was plotted as maximum increase in fluorescence (ΔF/F) after injection of capsaicin. FSK-potentiated capsaicin responses were inhibited by morphine (1 μM), while capsaicin responses in the absence of FSK were not affected by morphine. **B **and **C, E **and **F**: Representative Ca^2+ ^responses evoked by capsaicin (300 nM) in FLAG-MOP/TRPV1 expressant 21 (**B **and **C**) and 13 (**E **and **F**). FLAG-MOP/TRPV1 colony 21 and 13 responded to injection with 300 nM capsaicin with a characteristic increase in Ca^2+^, represented as an increase in ΔF/F (**B **and **E**). Pre-incubation with morphine (1 μM) did not affect the magnitude of Ca^2+ ^responses (**B **and **E**). Pre-incubation with FSK (50 μM) and IBMX (100 μM) increased capsaicin-evoked Ca^2+ ^responses (**C **and **F**) and treatment with morphine (1 μM) inhibited FSK-potentiated capsaicin responses (**C **and **F**). Data are presented as mean ± SEM with n = 8 for each colony and capsaicin concentration and statistical significance were determined at peak Ca^2+ ^responses after addition of capsaicin. * *p *< 0.05 compared to FSK alone.

Having identified that FSK potentiates TRPV1 responses in our heterologous expression system, we assessed whether morphine inhibits basal or FSK-potentiated TRPV1 responses to capsaicin. This was determined using Fluo-3 loaded FLAG-MOP/TRPV1 HEK293 colonies pre-incubated for 15 min with morphine (1 μM) for opioid-treated cells or PSS for non-opioid treated cells, followed by 15 min pre-incubation with FSK (50 μM) and IBMX (100 μM) for stimulated cells or PSS for unstimulated cells. Ca^2+ ^responses after injection of various capsaicin concentrations were monitored for 45 s. Pre-incubation of FLAG-MOP/TRPV1 colonies with morphine concentrations shown to be effective at reducing cAMP accumulation (Figure 3) did not affect the capsaicin dose-response curve for colonies 13 and 21 for unstimulated responses (*p *> 0.05; Figure 5A,B,D and 5E). However, pre-incubation with morphine significantly altered the capsaicin dose-response curve in FSK/IBMX stimulated cells (*p *< 0.05; Figure 5).

**Figure 6:**
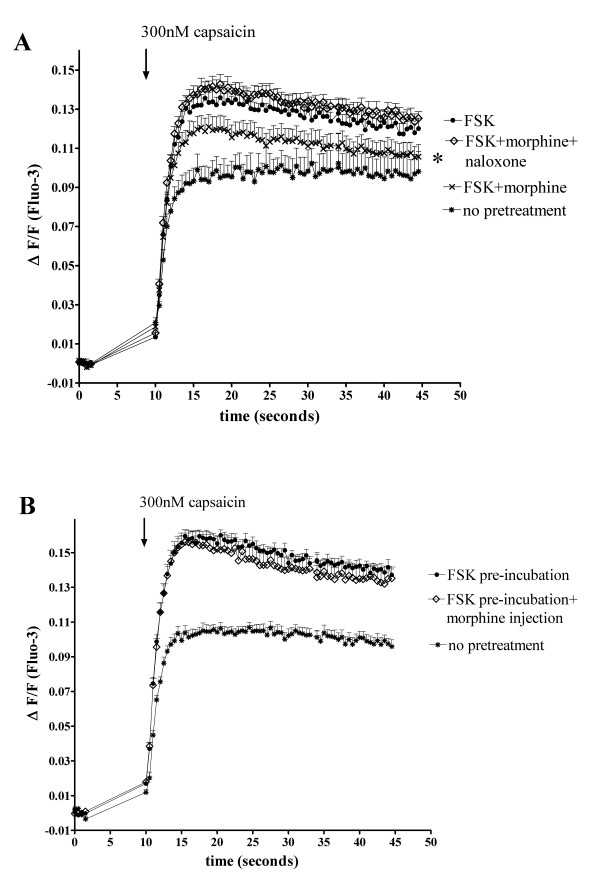
(**A**) Naloxone reverses morphine-inhibition of FSK-potentiated TRPV1 Ca^2+ ^responses. Fluo-3 loaded FLAG-MOP/TRPV1 double expressants (Colony 21) were pre-incubated with PSS for untreated cells (*) or morphine (1 μM) and naloxone (50 μM) as appropriate for treated cells for 15 min, followed by incubation with PSS for unstimulated cells or FSK (50 μM) and IBMX (100 μM) for stimulated cells. Pre-treatment with the opioid receptor antagonist naloxone (50 μM) (◊) prevented inhibition of FSK-potentiated capsaicin (300 nM) responses (●) by morphine (**x**). (**B**) Pre-incubation with morphine and activation of second messenger systems is required for morphine-inhibition of FSK-potentiated capsaicin responses. Fluo-3 loaded FLAG-MOP/TRPV1 double expressants (Colony 21) were pre-incubated with PSS for unstimulated cells (*) or FSK (50 μM) and IBMX (100 μM) for stimulated cells. Injection of morphine (◊) to a well concentration of 1 μM alongside capsaicin (300 nM) did not affect capsaicin (300 nM) responses in FSK-stimulated cells (●). Data are presented as mean ± SEM for n = 8 and statistical significance was determined at peak Ca^2+ ^responses after addition of capsaicin. * p < 0.05 for FSK+morphine compared to FSK alone.

The inhibition of FSK-stimulated capsaicin (300 nM) responses by morphine occurred through a MOP-specific pathway as the opioid receptor antagonist naloxone (50 μM) blocked inhibition by morphine (1 μM) (*p *< 0.05 compared to FSK+morphine and *p *> 0.05 compared to FSK; Figure 6A).

Since morphine-inhibition of FSK-stimulated capsaicin responses could be due to a reduction in cAMP level induced by treatment with opioid agonist, we assessed whether pre-incubation with morphine was required to achieve modulation of FSK-potentiated capsaicin responses. To do this, 300 nM capsaicin alone or 300 nM capsaicin with morphine (final well concentration of 1 μM) in cells were added to FLAG-MOP/TRPV1 HEK293 cells pre-incubated with FSK (50 μM) and IBMX (100 μM). Pre-incubation with morphine was required to achieve inhibition of FSK-stimulated capsaicin responses, as co-injection of morphine (1 μM) and capsaicin did not change the Ca^2+ ^response of FLAG-MOP/TRPV1 cells to 300 nM capsaicin (*p *> 0.05; Figure 6B).

### Morphine does not inhibit capsaicin responses potentiated by 8-Br-cAMP

**Figure 7:**
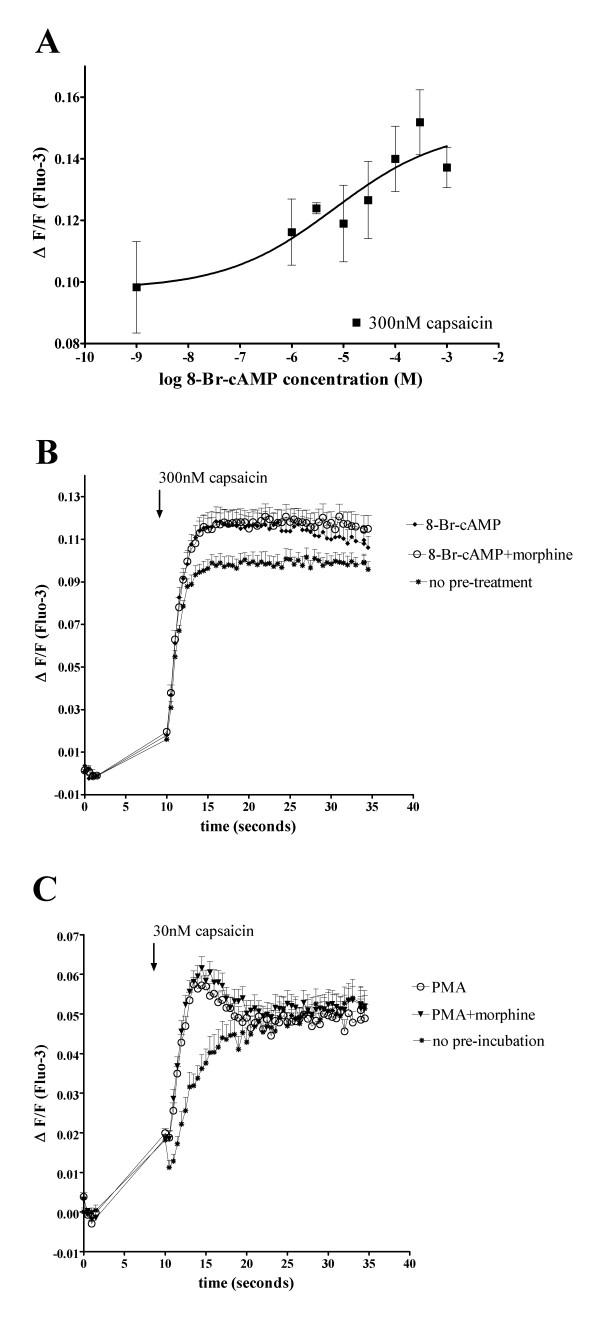
8-Br-cAMP stimulated capsaicin responses are not inhibited by morphine. (**A**) FLAG-MOP/TRPV1 double stable HEK293 cells (Colony 21) were loaded with the fluorescent Ca^2+ ^dye Fluo-3 and Ca^2+ ^responses to injection of 300 nM capsaicin were monitored using a fluorescent Microplate reader. Cells were pre-incubated for 15 min with IBMX (100 μM) as well as varying concentrations of the direct PKA activator 8-Br-cAMP (1 mM, 300 μM, 100 μM, 30 μM, 10 μM, 3 μM, 1 μM or PSS). Capsaicin (300 nM) was injected and Ca^2^+ responses plotted as the maximum increase in ΔF/F after capsaicin injection. Pre-incubation with 8-Br-cAMP and IBMX potentiated Ca^2+ ^responses to 300 nM capsaicin in an 8-Br-cAMP-concentration-dependent manner. (**B**) Morphine does not inhibit capsaicin responses potentiated by the direct PKA activator 8-Br-cAMP. Fluo-3 loaded FLAG-MOP/TRPV1 cells were incubated with PSS for untreated cell (◆) or morphine (1 μM) for treated cells (○) for 15 min, followed by potentiated with IBMX (100 μM) and 8-Br-cAMP (300 μM) for 15 min. Untreated control responses are denoted by (*). Pre-incubation with morphine did not inhibit 8-Br-cAMP potentiated capsaicin responses (○). Data are presented as mean ± SEM for n = 4–8. (**C**) Morphine does not inhibit capsaicin responses potentiated by the PKC activator PMA. Fluo-3 loaded FLAG-MOP/TRPV1 cells were incubated with PSS for untreated cell (*). PMA-potentiation was achieved by incubation of cells for 10 minutes with PMA (100 nM), preceded by 15 min incubation with PSS for control responses (○) or morphine (1 μM) for treated cells (▼). Pre-incubation with morphine did not inhibit 8-Br-cAMP potentiated capsaicin responses.

FSK is an indirect PKA activator, as it stimulates adenylate cyclase, which in turn leads to an increase in cellular cAMP levels, which activates cAMP-dependent PKA [13]. Several papers have reported potentiation or sensitization of TRPV1 responses through PKA-dependent pathways, including by treatment with indirect PKA activators like FSK or direct PKA activators such as 8-Br-cAMP [6,7,13]. The MOP is negatively coupled to adenylate cyclase and can inhibit FSK-stimulated cAMP production but would not affect direct PKA activators such as 8-Br-cAMP. To determine whether morphine-inhibition of FSK-stimulated capsaicin response relies on indirect inhibition of PKA through a decrease of cAMP levels, FLAG-MOP/TRPV1 double stable HEK293 cells were pre-incubated for 15 min with varying concentrations of 8-Br-cAMP as well as constant IBMX concentrations (100 μM) and Ca^2+ ^responses to 300 nM capsaicin were monitored (Figure 7A). Pre-incubation of FLAG-MOP/TRPV1 double expressants with 8-Br-cAMP (300 μM) and IBMX (100 μM) potentiated Ca^2+ ^responses to 300 nM capsaicin (Figure 7A &7B) in a manner that was dependent on 8-Br-cAMP concentration, as pre-incubation with IBMX alone did not significantly affect capsaicin responses (ΔF/F capsaicin 0.116 ± 0.004; ΔF/F capsaicin +IBMX 0.125 ± 0.003; *p *> 0.05).

Pre-incubation for 15 min with morphine (1 μM) did not (*p *> 0.05) affect Ca^2+ ^responses to injection of 300 nM capsaicin potentiated by 15 min pre-incubation with 8-Br-cAMP (300 μM) and IBMX (100 μM) (Figure 7B), suggesting that morphine inhibits FSK-potentiated capsaicin responses by indirectly inhibiting cAMP-dependent PKA.

### Morphine does not inhibit capsaicin responses potentiated by PMA

Several papers have reported potentiation of TRPV1 responses through PKC-mediated pathways [16,22,23], and PKC-potentiated TRPV1 responses have been shown to contribute to diabetic neuropathy [24]. Thus, in order to determine whether morphine can inhibit TRPV1 responses potentiated by other kinase pathways, we assessed inhibition of capsaicin responses by the PKC activator PMA.

Treatment with PMA (100 nM) significantly (p < 0.05) potentiated TRPV1-mediated capsaicin responses. MOP function, assessed by morphine inhibition of cAMP accumulation, was preserved in the presence of PMA (data not shown). Injection of PMA (10 μM) did not cause a Ca^2+ ^responses in FLAG-MOP/TRPV1 expressants (ΔF/F PSS 0.012 ± 0.004; ΔF/F PMA (10 μM) 0.016 ± 0.002; *p *> 0.05).

Pre-incubation for 15 min with morphine (1 μM) did not (*p *> 0.05) affect Ca^2+ ^responses to injection of 30 nM capsaicin potentiated by 10 min pre-incubation with PMA (100 nM) (Figure 7C).

### TRPV1 and MOP are co-expressed in cultured DRG neurones

**Figure 8:**
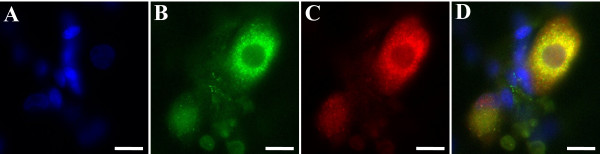
Cultured Dorsal Root Ganglion Neurones co-express TRPV1 and MOP. (**A**) DAPI stain (blue) shows cell nuclei of neurones as well as accessory cells. (**B**) Labelling with anti-MOP antibody, visualised with a FITC-conjugated secondary antibody, shows expression of MOP protein in cells with neuronal morphology, but not accessory cells. (**C**) TRPV1 protein (red) was visualised using a TRPV1 antibody with Cy3-conjugated secondary antibody. (**D**) Overlay of panels A, B and C shows co-expression of TRPV1 and MOP (yellow) in cultured DRG neurones. Scale bar = 12 μm.

To determine if a population of DRG neurons co-expressed MOP and TRPV1, immunohistochemical studies were performed. Nuclear labelling with DAPI showed that TRPV1 and MOP antibodies only labelled cells with neuronal morphology, while many cells showing DAPI labelling, presumably accessory cell types such as glia and astrocytes, remained unlabelled for TRPV1 and MOP (Figure 8A–C). Staining patterns for TRPV1 and MOP were in accordance with published literature [25-28].

Of the 173 cultured DRG neurones which represented the subset of neurons that were labelled for TRPV1 and MOP, 88.4% were co-labelled for TRPV1 (Figure 8C, red) and MOP (Figure 8B, green) with the overlay (yellow) clearly showing co-expression of the receptors, although the level of expression was somewhat variable between cells. Eleven neurones (6.4%) were labelled for TRPV1 only, while a further 9 neurones (5.2%) expressed only MOP.

## Discussion

The results reported in this study provide the first evidence for a functional interaction between the MOP and the TRPV1 and indicate that this interaction likely occurs via modulation of PKA-augmented TRPV1 responses. These results may have implications in inflammatory pain.

For both the MOP and TRPV1, transfected HEK-293 cells are a widely used model for assessing receptor functions as well as interactions with other receptors [12,16,28-31]. Using a heterologous expression system, we showed that morphine does not inhibit capsaicin-induced TRPV1 responses under basal conditions. However, morphine inhibits FSK-potentiated TRPV1 responses through action at the MOP receptor, as indicated by reversal with the opioid receptor antagonist naloxone. This inhibition is likely dependent upon modulation of cAMP-dependent PKA, as morphine does not affect capsaicin responses potentiated by the direct PKA activator 8-Br-cAMP or the PKC activator PMA.

The antinociceptive actions of opioids are more efficacious in inflammation; and MOP as well as TRPV1 expression are increased in inflamed tissues, along with increased cAMP levels in these tissues [9,32,33]. While binding affinities of MOP agonists remain unchanged, the efficacy of G-protein coupling is increased during inflammation [34]. In addition, opioid receptor expression is markedly upregulated as early as one day after induction of inflammation, and this increase in expression is paralleled by an increase in inhibition of neuronal Ca^2+ ^responses [34,35]. Due to disruption of the perineurium in inflammation, opioids have improved access to peripheral nerve endings on these inflamed tissues [36]. These changes highlight not only the importance of inflammatory sensitization in altering nociceptive receptor function but also the importance of sensitization in possibly increasing the ability of MOP to inhibit these sensitized inflammatory nociceptive responses. However, molecular pathways involved in this increased efficacy of opioids in inflammation have not been systematically assessed to date.

In peripheral sites of inflammation, opioids have been shown to inhibit neurogenic inflammation by decreasing the release of substance P from peripheral afferent terminals [37]. Notably, activation of the TRPV1 causes SP release and neurogenic inflammation [38] and opioids have been shown to inhibit capsaicin-evoked substance P release [39].

The specific MOP agonist endomorphine as well as the clinically useful MOP agonist morphine have been shown to produce anti-inflammatory effects in animal models and human studies when injected directly into inflamed tissues [40-44]. Importantly, activation of the TRPV1 by capsaicin induces hyperalgesia that can, dose-dependently and in a naloxone-sensitive manner, be inhibited by peripherally applied μ- and κ-opioid agonists [45-47], although the mechanisms involved in opioid-inhibition of TRPV1 responses have not been elucidated to date. Our results present a novel molecular basis for the anti-inflammatory action of peripheral opioids through a functional interaction of the MOP and PKA-sensitized TRPV1.

Several receptors have been reported to functionally interact with the TRPV1 [6,12,16,17,28,48]. Most of these receptors effect potentiation of TRPV1 responses through activation of second messenger systems, resulting in activation of PKC and cAMP-dependent PKA pathways [6,12,16,17,28,48]. Specifically, Insulin Growth Factor-1, insulin, bradykinin, nerve growth factor and prostaglandins can sensitize the TRPV1 through PKC-mediated phosphorylation, while PKA is involved in PGE_2_- and anandamide-mediated TRPV1 sensitization as well as potentiation of capsaicin responses through the metabotropic glutamate receptor 5 [6,16,17,28,48]. The PKA pathway has been proposed to be involved in the development of inflammatory hyperalgesia [9,11]. cAMP levels are elevated in inflamed tissues [9,10] and PKA or adenylate cyclase activators lower nociceptive thresholds while PKA inhibitors can be anti-hyperalgesic [49]. The cAMP/PKA pathway appears to be essential in sensitizing inflammatory nociception and contributes to the development of inflammatory hyperalgesia induced by proinflammatory mediators such as prostaglandin E2 (PGE_2_) [9,11].

Activation of peripheral group II metabotropic glutamate receptors inhibits potentiation of capsaicin responses by both PGE_2 _and FSK, but not potentiation by the direct PKA activator 8-Br-cAMP [14], consistent with our findings with MOP inhibiting TRPV1 in the presence of FSK. Although there are no previous studies of opioid receptors altering PKA-sensitization of capsaicin responses, the G-protein coupled cannabinoid receptor 1 (CB1) inhibits FSK-potentiated capsaicin responses in a PKA-dependent manner [12,50]. Importantly, while anandamide and other CB1 agonist can directly activate the TRPV1 under basal conditions and cause TRPV1-mediated Ca^2+ ^influx [12,50], our results showed that opioids did not affect capsaicin-induced TRPV1 responses under basal conditions. Our results suggest that activation of opioid receptors could be utilized to prevent sensitization of TRPV1 receptors by cAMP-dependent PKA in inflammation without the potential to activate unpotentiated TRPV1.

Activation of opioid receptors decreases cAMP levels and inhibition of NMDA currents by opioids occurs in a PKA-dependent manner through inhibition of adenylate cyclase [51]. Opioid modulation of TRPV1 responses may thus occur in inflamed tissues, where cAMP levels are elevated [11] through inhibition of adenylate cyclase and a subsequent inhibition of potentiation of TRPV1 responses by cAMP-dependent PKA. The results presented here support the hypothesis that the MOP and TRPV1 can functionally interact through a cAMP-dependent PKA pathway, as morphine inhibited FSK-potentiated capsaicin responses but not capsaicin responses in the absence of FSK. This is the first demonstration of a functional interaction between the TRPV1 and the MOP at the molecular level, and the interaction demonstrated here may be of significance in the treatment of inflammatory pain. In inflammation, when signalling through the TRPV1 is enhanced through increased PKA activity, activation of peripheral MOP could be utilised to prevent or modify TRPV1 sensitization. Moreover, while PKA pathways have been recognised to contribute to the development of inflammatory hyperalgesia, PKC pathways and in particular PKC-mediated phosphorylation have been postulated to contribute to the development of neuropathic hyperalgesia and allodynia [24,52]. We did not observe morphine modulation of PKC-potentiated TRPV1 responses; thus, the differential involvement of PKA and PKC in neuropathic and inflammatory pain may provide a mechanistic explanation for the predominant efficacy of morphine in inflammatory but not neuropathic pain states.

The time-dependency of morphine-inhibition of FSK-potentiated capsaicin responses supports the notion that, via activation of the MOP, and subsequent negative coupling to adenylate cyclase through G proteins [51], morphine can prevent sensitization of capsaicin responses through the cAMP/PKA pathway. cAMP assays performed on our FLAG-MOP/TRPV1 cell line showed that while incubation with morphine significantly reduced FSK-stimulated cAMP production, morphine at the concentrations used here, did not reverse cAMP levels to unstimulated levels. Accordingly, morphine did not completely inhibit FSK-potentiation of TRPV1-mediated Ca^2+ ^responses.

To rule out the possibility that morphine modulates FSK-potentiated capsaicin responses through action on phosphodiesterase or PKA and other downstream effectors, we used 8-Br-cAMP, a direct PKA activator, to potentiate capsaicin responses. While 8-Br-cAMP potentiated capsaicin responses similar to FSK, morphine did not modulate capsaicin responses potentiated by 8-Br-cAMP.

As the MOP is coupled negatively to adenylate cyclase and thus indirectly to PKA [18,51], direct activation of PKA and thus potentiation of capsaicin responses would negate the inhibitory effect of opioids on adenylate cyclase. These results indicate that morphine modulates FSK-potentiated capsaicin responses by inhibiting adenylate cyclase.

Several studies show expression of the TRPV1 and MOP on peripheral neurones as well as DRG [53,54]. More specifically, expression of TRPV1 has been reported in a small subset of trk-A and IB4-positive DRG neurons [1,2,55]. In addition, the number of TRPV1-expressing neurons increased after inflammation [3], with NGF reportedly regulating TRPV1 expression in trk-A-positive neurons [55].

While the DRG culture conditions utilised in our study included NGF and may therefore represent increased expression of TRPV1 in trk-A-expressing fibres, our findings are nonetheless relevant as increased NGF levels occur physiologically during inflammation [55]. Our finding of significant co-expression of TRPV1 and MOP in cultured DRG neurons was further confirmed in a recently published study assessing TRPV1 and MOP expression in both L4 and L5 dorsal root ganglia as well as lumbar spinal cord of rats [56]. While the proportion of neurons expressing TRPV1 and MOP was not quantified, similar to our results most neurons appeared to co-express TRPV1 and MOP with only relatively few cells expressing only one of the two receptors [56]. In addition, comparable to the percentage of neurons co-expressing TRPV1 and MOP in our study, Sanderson Nydahl et al reported 83% of L4 and L5 DRGs to co-express the MOP agonist endomorphin and TRPV1 [57], This co-expression of TRPV1 and MOP in the majority cultured DRG neurones may provide the anatomical basis for functional interaction of the nociceptive TRPV1 and antinociceptive MOP receptors, thus allowing for local regulation and subsequent modification of nociception.

However, inhibition of capsaicin-induced hyperalgesia in vivo could rely not only on PKA mediated pathways but may also include inhibition of Ca^2+ ^channels or the inhibition of substance P release [39].

## Conclusion

Our studies demonstrate for the first time that in a heterologous expression system where MOP and TRPV1 are stably co-expressed, morphine can inhibit capsaicin responses when the cAMP pathway is activated and this occurs through opioid-modulation of adenylate cyclase and thus indirectly PKA-mediated TRPV1 sensitization. As TRPV1 is expressed peripherally [3,4] and PKA-mediated sensitization occurs in these peripheral nociceptors via inflammatory mediators [9], targeting of non-central opioid receptors in inflammatory conditions may prevent peripheral sensitization and thus contribute to analgesia. Moreover, increased MOP expression and function during inflammation may contribute to local regulation of sensitization of nociceptors. This interaction may present the molecular basis for endogenous anti-inflammatory effects of opioids.

## Materials and methods

### Materials

Capsaicin and capsazepine were purchased from Sigma Aldrich (Castle Hill, NSW, Australia) and prepared as stock solutions in ethanol. Maximum final ethanol concentrations did not exceed 0.001%. 3-Isobutyl-1-methyl-xanthine (IBMX), forskolin (FSK), 8-bromo-adenosine 3',5'-cyclic monophosphate (8-Br-cAMP) and Phorbol 12-myristate 13-acetate(PMA) were from Sigma Aldrich and prepared as stocks in 100% dimethylsulfoxide (DMSO), with maximum DMSO concentrations not exceeding 0.1%. Morphine was obtained from David Bull Laboratories (Mulgrave, Victoria, Australia) and diluted in physiological salt solution (PSS). Geneticin and hygromycin selection antibiotics were from Invitrogen (Mulgrave, Victoria, Australia). QIAEX II agarose gel extraction kit was purchased from Qiagen (Doncaster, Victoria, Australia). Polyclonal rabbit anti-FLAG M2 antibody was purchased from Sigma. Polyclonal guinea-pig anti-MOP and polyclonal rabbit anti-TRPV1 antibodies were from Chemicon (Boronia, Victoria, Australia). Cy3-conjugated goat anti-mouse antibody was from Zymed Laboratories (San Francisco, CA) and FITC-conjugated goat anti-guinea pig as well as FITC-conjugated anti-mouse antibodies were purchased from Sigma. Slow-Fade Antifade was from Molecular Probes (Eugene, OR). Lipofectamine 2000 was purchased from Invitrogen (Mulgrave, Victoria, Australia). Protein estimation kit and Mini Trans-Blot^® ^Electrophoretic Transfer Cell were purchased from Bio-Rad (Hercules, CA). Enhanced chemiluminescence Plus (ECL Plus) was obtained from Amersham Biosciences (Buckinghamshire, England), and cAMP kits were supplied by Cayman Chemicals (Ann Arbor, MI). All other chemicals were purchased from Sigma.

### Construction of the pcDNA3.1 Hygro(+)-TRPV1 plasmid

The pcDNA3 plasmid containing the cDNA of the rat TRPV1 was a kind gift from David Julius (University of California, San Francisco) [1]. The TRPV1 sequence was released from the pcDNA3 plasmid using KpnI and XbaI, isolated with QIAEX II agarose gel extraction kit and subcloned into the plasmid pcDNA3.1 Hygro (+) (Invitrogen), which contains a hygromycin resistance sequence, using the corresponding KpnI and XbaI sites. The nucleotide sequence of the subcloned TRPV1 was compared against GeneBank.

### Generation of a HEK293 cell line stably expressing MOP and TRPV1

A plasmid containing the cDNA encoding the murine FLAG-epitope-tagged μ opioid receptor (FLAG-MOP) as well as a geneticin-resistance gene was a generous gift from Dr Selena Bartlett (Ernest Gallo Clinic and Research Centre, San Francisco, California).

In brief, HEK293 cells were transfected with pcDNA3 containing FLAG-MOP using Lipofectamine 2000 (Invitrogen) according to the manufacturer's protocol and stable FLAG-MOP expressants were selected with geneticin (0.5 mg/ml). Colonies originating from a single cell were selected and FLAG-MOP expression verified using immunohistochemistry and Western Blotting and a single clone was selected for transfection of the TRPV1. In a similar manner the pcDNA3.1 Hygro (+)/TRPV1 was transfected into the stable FLAG-MOP expressant. In order to select cells stably expressing functional TRPV1, cells were selected using hygromycin (75 μg/ml) and colonies of approximately 500 cells were isolated, plated on 96 well plates and further screened for functional TRPV1 expression by measuring Ca^2+ ^responses after injection of capsaicin (10 μM). TRPV1 expression was verified by Western blotting. Double stable FLAG-MOP/TRPV1 expressants were split every 4–6 days using trypsin/EDTA and maintained at 37°C in a 5% humidified CO_2 _incubator in Dulbecco's modified Eagle's medium (DMEM) containing 10% heat-inactivated foetal bovine serum (FBS), 2 mM L-glutamine, pyridoxine and 110 mg/ml sodium pyruvate supplemented with geneticin (0.2 mg/ml) and hygromycin (50 μg/ml).

### SDS-PAGE and Western Blotting

For detection of MOP and TRPV1 receptor proteins, stable FLAG-MOP/TRPV1 double expressants were plated on 10 cm dishes and grown to confluency before sample preparation with ice cold lysis buffer (NaCl 100 mM; Igepal 1%, Na deoxycholate 0.5%; Tris pH 8.0 50 mM) containing complete protease inhibitor mix (Roche, Castle Hill, NSW). Cells were removed from the culture dish with lysis buffer, homogenised by vortexing and incubated for 20 min on ice. After centrifugation at 6000 × g at 4°C for 20 min, protein estimation of the supernatant was performed according to manufacturer's instructions using a protein estimation kit (BioRad). Protein (20 μg) was precipitated by boiling at 90°C for 2 min and separated on a 7.5% SDS PAGE gel. Proteins were transferred to nitrocellulose membrane and blocked overnight at 4°C in blocking buffer (130 mM NaCl, 2.7 mM KCl, 10 mM NaH_2_PO_4_, 1.8 mM KH_2_PO_4_, 0.1% Tween-20 and 5% low-fat skim milk powder), followed by incubation for 1 h with anti-TRPV1 antibody (1:1000) for detection of TRPV1 protein or anti-FLAG M2 antibody (1:330) for detection of FLAG-MOP protein. For TRPV1 immunoblots, an anti-β-actin antibody (1:10 000) was also included for visualisation of β-actin to serve as a loading control (Data not shown). After a wash with blocking buffer, blots were incubated with appropriate secondary horseradish peroxidase (hrp)-conjugated antibody (1:5000; Zymed) in blocking buffer for 1 h and processed using ECL Plus for visualization by exposure to Eastman Kodak XAR film.

### cAMP accumulation assay

For the cAMP accumulation assay, FLAG-MOP/TRPV1 double expressants were plated on 12 well plates and grown to 90% confluency. Twenty-four hours prior to sample preparation, media was changed to DMEM supplemented with 0.5% heat-inactivated FBS. On the day of sample preparation, cells were washed with DMEM to remove serum and incubated with serum-free DMEM containing the phosphodiesterase inhibitor IBMX (100 μM) for 30 min, to which morphine (1 μM) was then added and cells incubated for a further 15 min. Following this, FSK (50 μM) was added to the wells and the cells incubated for 15 min to stimulate cAMP production. DMSO alone was used as a vehicle control. After incubation, reactions were terminated by aspiration of the medium and addition of 0.1 M HCl followed by 20 min incubation at room temperature. After centrifugation of the cell samples at 10,000 × g for 10 min, the protein content of the supernatant was assessed and the samples were diluted to protein concentrations of 20 μg/ml using enzyme immunoassay (EIA) buffer supplied with the cAMP EIA kit. Intracellular cAMP levels were measured with a competitive cAMP EIA kit in triplicate as per manufacturer's instructions.

### Microplate reader measurement of intracellular Ca^2+ ^responses in FLAG-MOP/TRPV1 HEK293 cells

Ca^2+ ^responses of FLAG-MOP/TRPV1 HEK293 cells were assessed using the fluorescent Ca^2+ ^probe Fluo-3. FLAG-MOP/TRPV1 expressants were plated on poly-D-lysine (PDL)-coated 96-well plates at a cell density of approximately 3–4 × 10^5 ^cells/ml 3 to 5 days prior to experiments and used at 70–90% confluency. Cells were loaded with Fluo-3 AM (6 μM) in loading buffer (pH 7.4, composition as PSS plus 3 mg/ml bovine serum albumin (BSA)) for 20–30 min at 37°C. Wells containing FLAG-MOP/TRPV1 double expressants were then washed three times with PSS (pH 7.4, composition KCl 5.9 mM, MgCl_2 _1.5 mM, NaH_2_PO_4 _1.2 mM, NaHCO_3 _5.0 mM, NaCl 140.0 mM, Glucose 11.5 mM, CaCl_2 _1.8 mM and HEPES 10.0 mM) and incubated for 15 min with PSS or opioid followed by 15 min of incubation with PSS or kinase activators with opioid as appropriate. Pre-incubation steps and Ca^2+ ^imaging was carried out at 29°C in order to avoid subcellular dye compartmentalization while maintaining constant experimental conditions. Capsaicin and other drugs were injected from a 10 × concentrated stock in order to achieve the required well concentration.

Fluo-3 loaded cells were excited at 420 nm and emission was recorded at 510 nm using a NOVOstar fluorescence microplate reader (BMG Victoria, Australia). Fluorescent emission readings were recorded every 0.5 s. Raw fluorescence data were corrected by subtracting the average fluorescence from 4 recordings just prior to addition of agonist from all subsequent time points. This value was then divided by the average fluorescence prior to addition of agonist to yield ΔF/F values [58-60]. For dose-response curves, maximum ΔF/F values from the response after addition of agonist were plotted against agonist concentration and a 4-parameter logistic Hill equation was fitted to the data using GraphPad Prism (San Diego, California). IBMX (100 μM) was contained in all experiments utilizing FSK and 8-Br-cAMP as well as in appropriate controls to exclude any effect of morphine on IBMX. All experiments were designed to include control experiments on the same plate as treated cells.

### Dorsal Root Ganglion (DRG) cell culture

DRGs were collected from adult male Wistar rats (200–350 g). The animals were kept in a controlled environment at a temperature of 22 ± 0.5°C, relative humidity of 40–60% and a 12 h (6 am–6 pm) light-dark cycle with free access to standard lab chow and tap water. Animals were sedated by inhalation of 50% O_2_/50% CO_2 _and sacrificed by asphyxiation with 100% CO_2_. DRGs were collected from the rat spinal cord under sterile conditions from level T12 to S2. Harvested DRGs were dissociated in media containing trypsin (0.5 mg/ml), collagenase (1 mg/ml) and DNAse (1 μg/ml) and incubated at 37°C and 5% CO_2 _for 45 to 60 min. Trypsin-chymotrypsin inhibitor (0.1 mg/ml) was then added and cell suspension centrifuged at 200 rpm for 10 min. The cell pellet was resuspended in 2 ml of warm medium consisting of DMEM supplemented with glutamine (4 mM), 10% horseserum, 10% foetal bovine serum, penicillin streptomycin (2 mM) and NGF (nerve growth factor) (100 ng/ml). Cells were plated on 25 mm PDL-coated glass coverslips at a density of 75,000 to 200,000 cells per coverslip. After 1 h, the medium was changed to Neurobasal-A medium supplemented with glutamine (4 mM), NGF (100 ng/ml) and B27 (20 μl/ml). Media was replaced every 2 days and experiments were performed after 3–6 days in culture. Cell cultures were confirmed visually to have characteristic DRG neuronal morphology combined with positive staining for synaptophysin.

### TRPV1 and MOP receptor immunoreactivity in cultured DRG neurones and FLAG-MOP/TRPV1 transfected HEK293 cells

Cells plated on 25 mm coverslips were washed twice in PBS (mM: NaCl 137; KCl 2.7; NaH_2_PO_4 _10; KH_2_PO_4 _1.8) for 10 min before being fixed with 4% paraformaldehyde for 20–30 min at room temperature. Cells were permeabilized with 0.2% Triton X in PBS for 10 min and blocked by immersion in PBS with 3% BSA for 30 min. Cells were washed thoroughly with PBS between all incubation steps. All antibodies were prepared in PBS containing 3% BSA. For DRG neurones, guinea pig anti-MOP primary antibody (1:5000) was applied for 1 h, while for FLAG-MOP/TRPV1 transfected HEK cells, mouse anti-FLAG M2 primary antibody (1:500) was applied for 1 h, followed by FITC-labelled anti-guinea pig or FITC-labelled anti-mouse secondary antibody (1:300) as appropriate in low light for 30 min. Cells were then incubated with rabbit anti-rat TRPV1 primary antibody (1:4000) for 1 h, followed by incubation with anti-rabbit Cy3-conjugated secondary antibody (1:300) under low light for 30 min. Subsequent incubation of coverslips with 4',6-diamidino-2-phenylindole dihydrochloride (DAPI; 300 nM) for 1–5 min was utilized to visualize cellular DNA. Specificity of the TRPV1 antibody was previously demonstrated [54] and confirmed here through lack of non-specific binding in cultured DRG neurones as well as double stable HEK293 cells (Figure 1F and 1G). Specificity of the MOP antibody was demonstrated by dual labelling of stable FLAG-MOP expressants with anti-MOP and anti-FLAG M2 antibodies, which showed excellent overlay (data not shown). Cells were thoroughly rinsed with PBS and distilled water and mounted using SlowFade AntiFade before viewing randomly selected fields of view on a Nikon Eclipse TE 300 inverted fluorescent microscope (excitation 488 nm, emission 520 nm for FITC; excitation 540 nm, emission 570 nm for Cy3 and excitation 364 nm, emission 454 nm for DAPI). Pictures were recorded using MetaFluor (Molecular Devices, Downington, PA) imaging software and processed using Paintshop Pro for Windows (Microsoft).

### Data analysis

Data are presented as mean ± standard error of the mean (SEM) of at least two to three independent experiments. Fitting of 4-parameter Hill equations and statistical analysis were carried out using GraphPad Prism Version 4 (San Diego, CA). Statistical significance was determined using an unpaired, two-tailed Student's t-test unless otherwise stated with statistical significance defined as *p *< 0.05.

## Abbreviations

8-Br-cAMP, 8-bromoadenosine 3'5'-cyclic monophosphate; BSA, bovine serum albumin; Ca^2+^, calcium ion; cAMP, cyclic AMP; DAPI, 4'6-diamidino-2-phenylindole dihydrochloride; DMEM, Dulbecco's modified eagles medium; DMSO, dimethylsulfoxide; DRG, dorsal root ganglion; EIA, enzyme immunoassay; Fluo-3 AM, Fluo-3 acetoxymethylester; FSK, forskolin; HEK293, human embryonic kidney cells; IBMX, 3-isobutyl-1-methyl-xanthine; MOP, μ opioid receptor; NGF, nerve growth factor; NMDA, N-methyl-D-aspartate; PBS, phosphate buffered solution; PDL, poly-D-lysine; PGE_2_, prostaglandin E2; PKA, protein kinase A; PKC, protein kinase C; PSS, physiological salt solution; TRPV1, transient receptor potential vanilloid receptor 1.

## Competing interests

The author(s) declare that they have no competing interests.

## Authors' contributions

IV carried out generation of TRPV1-transfected cell lines and associated experiments and drafted the manuscript. BW generated MOP-transfected HEK293 cells and aided with manuscript review. GM aided in presentation of Ca^2+ ^imaging data and manuscript presentation. SRT contributed to generation of the TRPV1-pcDNA3.1 Hygro+ plasmid, molecular biology work and proof-reading of the manuscript. PC contributed to the design and coordination of the study and helped to draft the manuscript. All authors read and approved the final manuscript.
